# Genetically Related Avian Influenza H7N9 Viruses Exhibit Different Pathogenicity in Mice

**DOI:** 10.3390/ani13233680

**Published:** 2023-11-28

**Authors:** Xiaoquan Wang, Huafen Zheng, Ruyi Gao, Leyao Ren, Mingxia Jin, Zhuxing Ji, Xin Wang, Xiaolong Lu, Wenhao Yang, Min Gu, Xiaowen Liu, Shunlin Hu, Kaituo Liu, Xiufan Liu

**Affiliations:** 1 College of Veterinary Medicine, Yangzhou University, Yangzhou 225009, China; 2 Jiangsu Co-Innovation Center for Prevention and Control of Important Animal Infectious Diseases and Zoonosis, Yangzhou University, Yangzhou 225009, China; 3 Jiangsu Key Laboratory of Zoonosis, Yangzhou 225009, China; 4 Joint International Research Laboratory of Agriculture and Agri-Product Safety, The Ministry of Education of China, Yangzhou University, Yangzhou 225009, China

**Keywords:** H7N9, pathogenicity, PB2-E627K, mice

## Abstract

**Simple Summary:**

Avian influenza viruses have the ability to breach species barriers and infect mammals, posing a significant threat to public health. The H7N9 subtype of avian influenza virus that emerged in China in 2013 resulted in 1568 human infections, with an alarming mortality rate of nearly 40%. While human infections caused by avian influenza viruses typically occur sporadically, the reasons behind the widespread impact of H7N9 remain unclear. During surveillance for avian influenza in live poultry markets in eastern China in 2013, we isolated two H7N9 avian influenza viruses from seemingly healthy chickens. One of these chicken-derived viruses demonstrated the ability to bind to mammalian receptors and naturally harbored the mammalian molecular marker PB2-627K. Furthermore, this virus proved to be highly pathogenic to mice. In summary, our findings suggest that the presence of a mammal-adapted H7N9 virus in poultry in 2013 could be a significant factor explaining the unusually high number of human infections during that period.

**Abstract:**

Avian influenza viruses can cross species barriers and adapt to mammals. The H7N9 subtype AIV that emerged in China in 2013 caused 1568 human infections, with a mortality rate of nearly 40%. We conducted a retrospective analysis of H7N9 viruses that were isolated in live poultry markets in 2013. We found that two avian-origin H7N9 isolates, A/chicken/Eastern China/JTC4/2013 and A/chicken/Eastern China/JTC11/2013, have a similar genetic background but exhibit different pathogenicity in mice. Whole-genome alignment of the two H7N9 viruses was carried out, and only six amino acid differences mapped in five genes, including the well-known virulence molecular marker PB2-E627K. Our retrospective analysis highlighted the importance of monitoring the adaptive mutations in avian influenza viruses with zoonotic potential.

## 1. Introduction

Influenza A viruses (IAVs) are single-stranded negative-sense RNA viruses that belong to the family of orthomyxoviruses. The IAV genome is prone to gene reassortment and is subdivided into 18 different hemagglutinin (HA) and 11 neuraminidase (NA) subtypes based on the surface proteins [1,2]. Different subtypes of IAVs usually have hosts of different natures [3,4,5]. Based on their natural host, IAVs can be divided into human viruses (H1N1, H2N2, and H3N2), avian influenza viruses (H1-H16 and N1-N9), swine influenza viruses (H1N1/2, H2N3, and H3N2), canine influenza viruses (H3N2 and H3N8), equine influenza viruses (H3N8 and H7N7), and bat influenza viruses (H17N10 and H18N11) [6]. Among them, avian influenza virus (AIV) contains the most extensive subtypes and can cross species barriers to infect mammals, including humans [6].

In recent years, multiple subtypes of AIVs have been reported to frequently infect humans, including H5N1, H5N6, H5N8, H7N9, H7N4, H10N8, H10N3, and H3N8 [7,8,9,10,11,12,13,14]. Among them, the H7N9 subtype AIV was the one that attracted the most attention of the public. Since the first case of human infection with H7N9 was found in China, five epidemic waves of human infection have occurred and resulted in 1568 infection cases, of which 615 cases were fatal [15,16].

Much of the research indicates that AIV’s adaptation to mammals occurs gradually [17,18,19]. Cross-species transmission is the result of many factors. The first step in the viral lifecycle is attachment to a susceptible host epithelial cell. Hemagglutinin (HA) is responsible for host receptor binding and is the primary determinant of the AIV’s host “jump” [20,21]. Following entry into the cell, the key factor determining the pathogenicity is effective viral replication. In general, early epidemic AIVs commonly do not replicate efficiently in mammalian cells. The viral polymerase complex is a major determinant of host range restriction [6,22]. With reference to avian H7N9 subtype AIVs, a series of molecular markers associated with mammalian adaptation were identified [23,24,25,26,27,28,29]. The discovery of these molecular markers could help in evaluating the threat of H7N9 to humans.

In this study, we performed a retrospective analysis of H7N9 AIVs that were isolated in live poultry markets in China in 2013. We found two H7N9 strains, A/chicken/Eastern China/JTC4/2013 (hereafter JTC4) and A/chicken/Eastern China/JTC11/2013 (hereafter JTC11), that had a similar genetic background, but the key molecular markers associated with pathogenicity in mammals were different. We fully evaluated the pathogenicity of this pair of strains in chickens and mice. The results showed that JTC4 and JTC11 were both associated with low pathogenicity in chickens. JTC4 was lowly pathogenic in mice; however, JTC11 exhibited high pathogenicity in mice. Our results showed that mammal-adapted H7N9 virus naturally existed in poultry in 2013.

## 2. Materials and Methods

### 2.1. Ethics Statement

The study was conducted in strict accordance with the guidelines outlined in the Guide for the Care and Use of Laboratory Animals by the Ministry of Science and Technology of the People’s Republic of China. All experiments involving live virulent H7N9 viruses and animals were carried out within the animal biosafety level 3 (ABSL-3) facilities at Yangzhou University, adhering to the recommendations specified in the institutional biosafety manual. The Committee for Laboratory Animals granted permission for these experiments (Permission numbers: SYXK-SU-2022-0044 and SYXK-SU-2021-0027), and the Institutional Biosafety Committee of Yangzhou University approved all procedures, ensuring compliance with the guidelines of the Jiangsu Laboratory Animal Welfare and Ethics of the Jiangsu Administrative Committee of Laboratory Animals.

### 2.2. Viruses

The two H7N9 avian influenza viruses (AIVs) investigated in this study, namely A/Chicken/Eastern China/JTC4/2013 and A/Chicken/Eastern China/JTC11/2013 (Table 1), were originally obtained from chickens in eastern China and stored in our laboratory. These viruses underwent three rounds of plaque purification in Madin–Darby canine kidney (MDCK) cells before being propagated into 9-day-old specific-pathogen-free (SPF) embryonated chicken eggs. The propagated viruses were then stored at −80 °C for future use. The nucleotide sequences of these two poultry-origin H7N9 viruses are accessible from the Global Initiative on Sharing All Influenza Data (GISAID) under accession numbers EPI2109797 to EPI2109812.

### 2.3. Genetic and Phylogenetic Analyses

Viral RNA was extracted from allantoic fluid using commercial kits (TransGen Biotech, Beijing, China) in accordance with the manufacturer’s instructions. The extracted RNA was then transcribed into cDNA using the primer Uni12 (5′-AGCAAAAGCAGG-3). Subsequently, eight segments of these viruses were amplified via PCR with specific primers. Positive PCR products were purified from the gel using the QIAquick PCR purification kit (QIAGEN, Valencia, CA, USA) and subjected to sequencing using the dideoxy method (Applied Biosystems, Shanghai, China). DNA sequences were compiled and edited using Lasergene 7.1 (DNASTAR, Madison, WI, USA). The reference sequences were downloaded from the online databases of the NCBI and GISAID. Phylogenetic trees of all eight genes were constructed employing the maximum likelihood method with bootstrap analysis (1000 replicates) using MEGA 7 software (Sinauer Associates, Inc., Sunderland, MA, USA).

### 2.4. Receptor Binding Properties

The receptor-binding specificity of H7N9 was assessed through hemagglutination assays using goose red blood cells (GRBCs) treated with an α-2,3-specific sialidase (Takara, San Jose, CA, USA). Solid-phase direct binding assays were conducted utilizing synthetic sialylglycopolymers, specifically Neu5Aca2-3Galb1-4GlcNAcb (3′SLN)-PAA-biotin and Neu5Aca2-3Galb1-4GlcNAcb (6′SLN)-PAA-biotin (GlycoTech), as previously described [14]. Poultry isolate A/mallard/Huadong/S/2005[HD05(H5N1)], exhibiting specificity for SAα-2,3 Gal, and a human isolate A/California/04/2009[CA04(H1N1)], demonstrating specificity for SAα-2,6 Gal, were employed as controls, respectively.

### 2.5. Virus Growth Kinetics

MDCK, human lung carcinoma (A549), and chicken embryo fibroblast (CEF) cell lines were infected with the two H7N9 viruses at a multiplicity of infection (MOI) of 0.001 and incubated at 37 °C in triplicate on 6-well plates. Cell supernatants were collected at 12 h intervals up to 72 h post-infection (hpi). Viral titers were determined by assessing the 50% tissue culture infectious dose (TCID_50_) per mL in MDCK cells. MDCK and A549 cells were obtained from the American Type Culture Collection (ATCC, Manassas, VA, USA). CEF was prepared from 9-day-old chicken embryos.

### 2.6. Animal Experiments

The 50% egg infectious dose (EID_50_) of each virus was determined in SPF eggs using the Reed and Muench method [30].

In accordance with the guidelines from the World Organization for Animal Health (OIE, 2018), ten six-week-old specific-pathogen-free (SPF) chickens per group (sourced from the Beijing Experimental Animal Center, Beijing, China) were intravenously inoculated with 0.1 mL of infectious allantoic fluid, at a dilution ratio of 1:10 in phosphate-buffered saline (PBS), to assess the corresponding intravenous pathogenicity index (IVPI).

Groups of five six-week-old female BALB/c mice (Yangzhou Experimental Animal Center, Jiangsu, China) were lightly anesthetized with pentobarbital sodium and intranasally inoculated with virus doses ranging from 10^3.0^ to 10^6.0^ EID_50_, each diluted in 50 µL PBS, for the assessment of the 50% mouse lethal dose (MLD_50_). Changes in body weight and mortality of the infected mice were monitored daily for a duration of 14 days. Mice were humanely euthanized if weight loss exceeded 25% of their initial weight, and they were considered dead for humane reasons.

To assess the tissue distribution of the virus in mice, groups of three mice were intranasally inoculated with 10^6.0^ EID_50_ of each virus in a 50 µL volume and euthanized at 3 days post-inoculation (dpi). Nasal turbinates, lungs, kidneys, and brains were collected and titrated by determining the EID_50_ in eggs. For histopathological analysis, mouse lungs collected at 3 dpi were fixed with 10% formalin, stained with hematoxylin and eosin (H&E), and examined under light microscopy.

### 2.7. Qualitative Analysis of Cytokines

Expression levels of interferon-beta (IFN-β), interleukin-beta (IL1-β), IL-6, inducible protein-10 (IP-10), interferon-stimulated gene 15 (ISG15), and Mx1 in the lung homogenates were qualitatively determined via quantitative real-time PCR (qRT-PCR). In brief, the lung homogenates were collected in the 10^6.0^ EID_50_ group at 3 dpi. The RNA was extracted according to the manufacturer’s instructions, and total RNA was reverse transcribed using the PrimeScript™ RT reagent Kit with gDNA Eraser. The extracted RNA was transcribed into cDNA, and the expression levels of each cytokine were determined using the TB Green premix Ex TaqTM II in a LightCycler480 System machine. Glyceraldehyde-3-phosphate dehydrogenase (GAPDH) expression was also detected and used for the normalization of gene expression between different samples.

## 3. Results

### 3.1. Genetic and Phylogenetic Analyses

**Figure 1:**
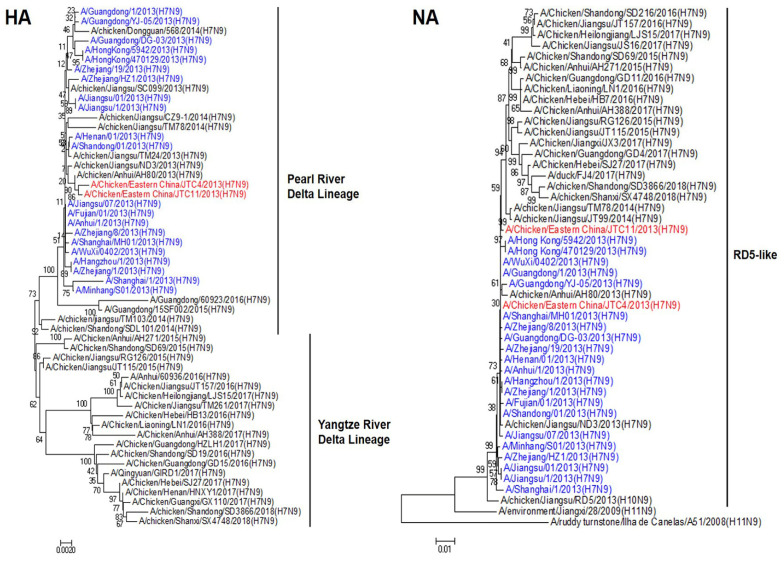
Phylogenetic trees of HA and NA of H7N9 viruses isolated from chicken in eastern China, 2013, with reference sequences. The two H7N9 viruses isolated in eastern China are highlighted in red, while human-origin H7N9 viruses isolated in China in 2013 are highlighted in blue. The neighbor-joining tree was generated using MEGA 7 software. The tree was constructed using the neighbor-joining method with the maximum composite likelihood model in MEGA version 7.0 (http://www.megasoftware.net, accessed on 12 September 2023) with 1000 bootstrap replicates.

**Figure 2:**
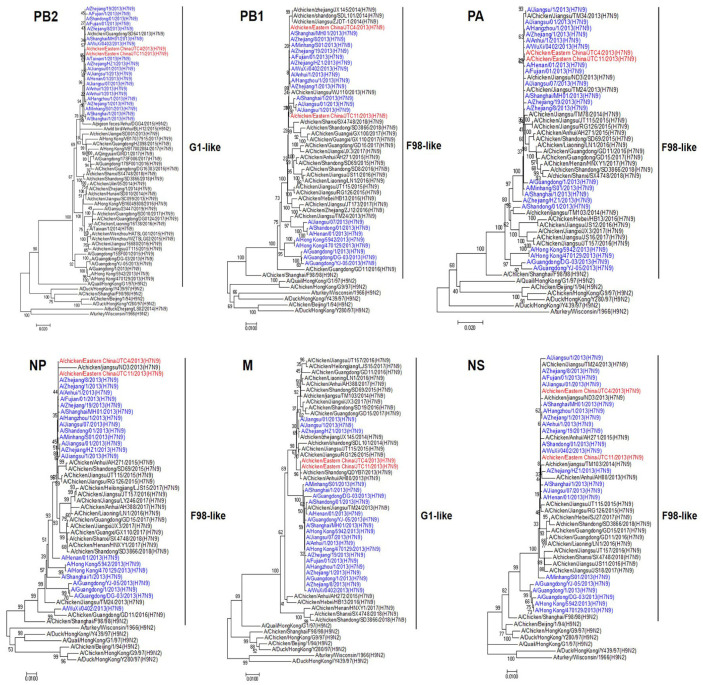
Phylogenetic trees of internal genes of H7N9 viruses isolated from chicken in eastern China, 2013, with reference sequences. The two H7N9 viruses from eastern China are highlighted in red, while H7N9 viruses of human origin isolated in China in 2013 are highlighted in blue. The neighbor-joining tree was generated using MEGA 7 software. Construction of the tree employed the neighbor-joining method with the maximum composite likelihood model in MEGA version 7.0 (http://www.megasoftware.net, accessed on 12 September 2023), and it included 1000 bootstrap replicates.

We sequenced all eight genes of the two chicken-origin H7N9 viruses to clarify their genetic properties. Phylogenetic analysis of the HA sequences revealed that they belonged to the Pearl River Delta Lineage and clustered with human-origin H7N9, which were isolated in China in 2013 (Figure 1). The NA gene of these two isolates had a high homologous rate with avian- and human-origin H7N9 and belonged to the RD5-like (H10N9) (Figure 1). Phylogenetic analysis of the internal genes revealed that the polymerase basic protein 2 (PB2), polymerase basic protein 1 (PB1), polymerase acidic protein (PA), nucleoprotein (NP), matrix protein (M), and nonstructural protein (NS) genes of the two poultry isolates were all sourced from genotype S (G57) H9N2 AIVs. These internal genes exhibited unique genetic constellations, specifically G1-like, F/98-like, F/98-like, F/98-like, G1-like, and BJ/94-like H9N2, respectively (Figure 2) [31,32]. These findings suggest that the two chicken-origin H7N9 isolates had a high homologous rate with avian- and human-origin H7N9.

### 3.2. Viral Growth Properties among Cell Lines

**Figure 3:**
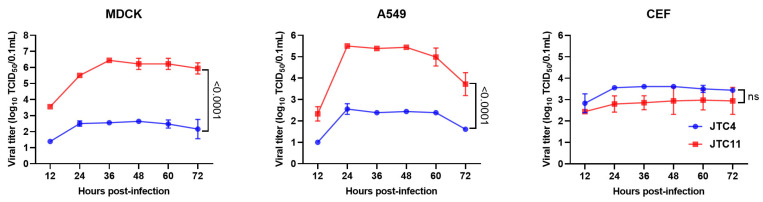
Viral growth properties of the two avian-origin H7N9 viruses in MDCK, A549, and CEF cell lines at a MOI of 0.001. The supernatants were monitored for virus titers using TCID_50_ in MDCK cells at the indicated time points. The values are expressed as means with standard deviations. Statistical analysis was performed using GraphPad software (9.3.0), and statistical significance was assessed using the two-tailed unpaired Student’s *t*-test. Statistical significance was reported at ns, *p* > 0.05.

The in vitro replication kinetics of the two viruses were measured in the MDCK, A549, and CEF cells. As shown in Figure 3, the two H7N9 viruses could effectively replicate in all three cell lines, and JTC11 yielded significantly higher titers than JTC4 in mammals’ cell lines (MDCK and A549) at all time points. JTC11 replicated with similar kinetics as JTC4 in the CEF cell, and the differences were not significant. Therefore, the replication abilities of the JTC11 virus were significantly higher than those of the JTC4 virus in mammalian cell lines.

### 3.3. Receptor Binding Properties

**Figure 4:**
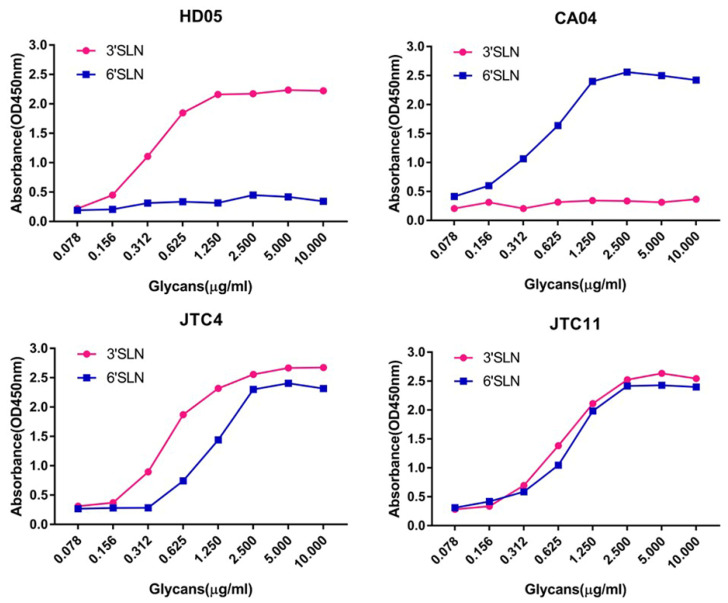
Receptor-binding properties of the two avian-origin H7N9 isolates. The control viruses, A/California/04/2009 (pdmH1N1) (CA04) and A/mallard/Huadong/S/2005 (H5N1) (HD05), exhibited a clear preference for human-type (SAα-2,6Gal) and avian-type (SAα-2,3Gal) receptors, respectively. The direct binding of viruses to sialylglycopolymers containing either 3′SLN-PAA or 6′SLN-PAA was quantified. The presented data are representative of three independent binding experiments.

The receptor-binding preference holds significant implications for assessing the interspecific transmission potential of AIVs [22,33]. To delineate the receptor-binding properties of the two H7N9 isolates, we conducted HA assays and solid-phase direct binding assays. A/mallard/Huadong/S/2005 (H5N1) and A/California/04/2009 (H1N1) were included as controls, demonstrating a robust affinity for avian-type receptors (SAα-2,3 Gal) and human-type receptors (SAα-2,6 Gal), respectively. The HA assays (Table 2) and solid-phase direct binding assays (Figure 4) showed that the two H7N9 isolates could bind to the SAα-2,3-Gal (avian-type) and SAα-2,6-Gal (human-type) receptors. This finding suggests that the two chicken-origin H7N9 isolates have the ability to infect birds and mammals.

### 3.4. Pathogenicity in Chickens

To investigate the pathogenicity in chickens, the two H7N9 viruses were intravenously inoculated to determine the IVPI. Chickens in both groups presented with no clinical symptoms and showed 100% survival during the 10 days, indicating that these viruses were lowly pathogenic in chickens (Table 1).

### 3.5. Pathogenicity in Mice

**Figure 5:**
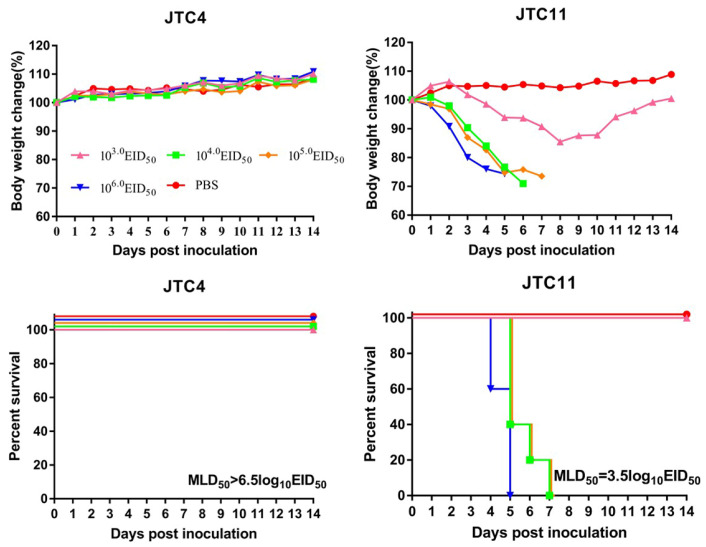
Virulence of the two avian-origin H7N9 isolates in mice. Groups of five 6-week-old female BALB/c mice were inoculated intranasally with 10^3.0^~10^6.0^ EID_50_ of JTC4 or JTC11 virus, respectively. Body weight and survival were monitored daily for 14 dpi; mice were humanely euthanized when they lost ≥25% of their initial body weight.

To investigate the pathogenicity in mammals, we evaluated the virulence of these two strains in BALB/c mice. As shown in Figure 5, none of the mice died in the JTC4-inoculated groups and even in the 10^6.0^ EID_50_ inoculation group. Therefore, the MLD_50_ for JTC4 was >6.5 log10 EID_50_. Mice inoculated with high doses of the JTC11 virus died during the observation period, yielding an MLD_50_ for JTC11 of 3.5 log10 EID_50_ (Table 1 and Figure 5).

**Figure 6:**
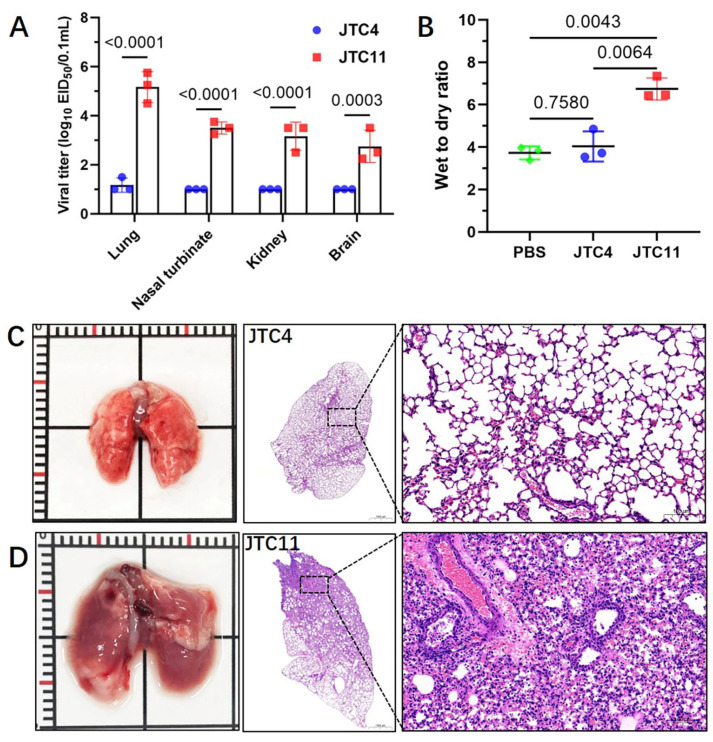
Replication and lung injury analysis of the two avian-origin H7N9 isolates in mice. Groups of three 6-week-old female BALB/c mice were inoculated intranasally with 50 µL of the specified viruses at a concentration of 10^6.0^ EID_50_. (**A**) At 3 dpi, viral loads in the nasal turbinate, lung, brain, and kidney were titrated in eggs, and the values are presented as means with standard deviations. Statistical analysis was conducted using GraphPad software, with significance assessed using the two-tailed unpaired Student’s *t*-test; (**B**) wet-to-dry ratio; lungs were collected at 3 dpi from mice and weights before and after drying were recorded; (**C**) JTC4 and (**D**) JTC11 underwent histopathological analysis at 3 dpi. Lungs collected from mice intranasally inoculated with 50 µL of the specified viruses at 10^6.0^ EID_50_ were fixed with formalin, embedded in paraffin, and stained with hematoxylin and eosin. Images were captured at magnifications of ×1.5 and 10.

To assess the replication capacities of viruses in mice, three infected mice in each group were euthanized at 3 dpi to determine viral titers in the lung, kidney, nasal turbinates, and brain. As shown in Figure 6A, JTC11 replicated efficiently in the nasal turbinates and lungs of mice, and virus was also detected in the kidney and brain. In contrast, only low doses of virus were detected in one mouse of the JTC4 group.

Pathological studies were conducted on the lungs of inoculated mice collected at 3 dpi. The H&E staining of the lungs of mice infected with JTC4 was morphologically normal (Figure 6C). JTC11-inoculated mice displayed thickening of the alveolar wall, severe inflammatory cell infiltration, hemorrhage, and bronchopneumonitis (Figure 6D).

**Figure 7:**
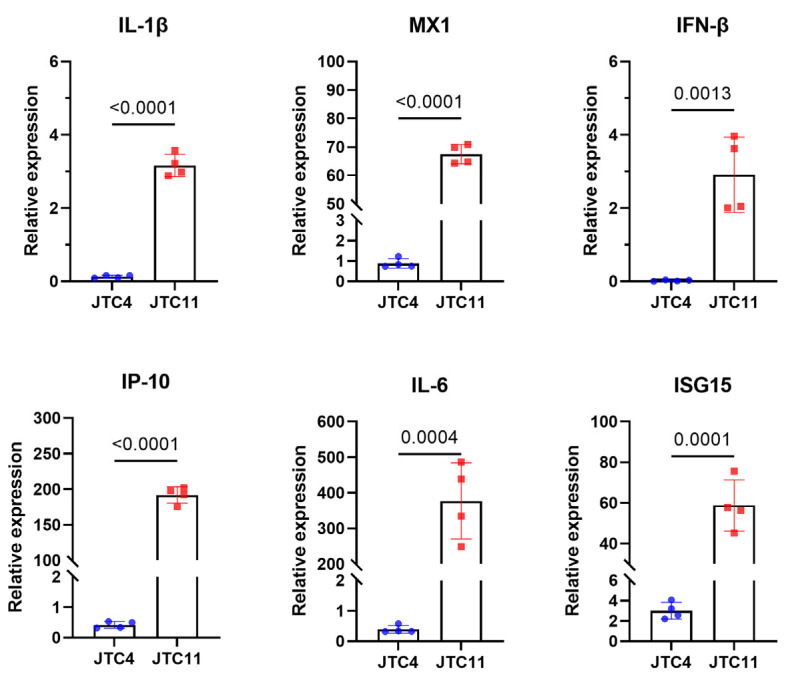
Inflammatory cytokine expression of the two avian-origin H7N9 isolates in mice. Mice were inoculated intranasally with 10^6.0^ EID_50_ of desired viruses; total RNA was extracted from lungs at day 3 post infection; equal amounts of RNA were used for qRT-PCR. The expression level of each cytokine gene was normalized to GAPDH and is presented as fold change compared with control. The values are expressed as means with standard deviations. Statistical analysis was performed using GraphPad software, and statistical significance was assessed using the two-tailed unpaired Student’s *t*-test.

It has been suggested that cytokine dysregulation is related to the enhanced pathogenicity of AIVs in mammalian hosts [34,35]. The wet-to-dry ratio results indicated that the lungs of the JTC11-inoculated mice experienced significant inflammatory exudates (Figure 6B). We then compared the induction of inflammatory cytokines in the lungs of mice infected with JTC4 and JTC11. The analysis of inflammatory cytokine expressions in the lung homogenates of mice showed the levels of pro-inflammatory cytokines (IL1-β, IL-6), chemokines (IP-10), and antiviral cytokine (IFN-β, Mx1, and ISG15) of JTC11-inoculated groups were significantly higher than those of the uninfected controls. Mice infected with the JTC4 virus only exhibited an increased expression of ISG15, while the levels of other cytokines were similar or lower than those of the control groups (Figure 7).

In summary, JTC4 exhibited low pathogenicity in mice. However, JTC11 caused mortality in mice, effectively replicated in multiple organs, caused severe inflammation, and increased the cytokine expression in the lungs of the mice.

### 3.6. Comparison of Whole-Genome Sequence between JTC4 and JTC11

In order to explore the reasons for the difference in pathogenicity in mice, whole-genome alignments of JTC4 and JTC11 were carried out. As shown in Table 3, JTC4 and JTC11 revealed six amino acid differences mapped in five genes containing PB2, PB1, PA, HA, and NA. It is worth noting that a well-known mammalian adaptation molecular marker was found in the JTC11 isolate, PB2-627K, which had fully demonstrated the ability to enhance AIV’s replication capacity and virulence in mammals [24,36,37].

## 4. Discussion

The last human case of H7N9 infection was reported in April 2019, which seems to signal that H7N9 is less of a threat to public health security in recent years [15]. Nevertheless, the H7N9 virus has not been eradicated from poultry [38,39,40]. In addition, H7N9 displayed antigenicity variation during circulation [38,41,42,43], which facilitates virus evasion of the immune response. Moreover, H7N9 also obtained a higher binding capability for α2,6-sialic acid receptors [39], retaining the ability to infect humans. Thus, it is necessary to maintain a high level of attention to H7N9 AIVs. In this study, we performed a retrospective analysis of H7N9 AIVs that were isolated in live poultry markets in 2013.

China has experienced five epidemics of the H7N9 virus since 2013 [15,44]. In the initial four epidemic waves, only low pathogenic avian influenza (LPAI) H7N9 viruses were in circulation, causing asymptomatic or mild disease in poultry [15]. Asymptomatic poultry play a pivotal role in the maintenance and amplification of the viruses, acting as sources of infection for other domestic poultry and migratory birds, thereby perpetuating and disseminating the viruses. In this study, the two poultry-origin H7N9 isolates were lowly pathogenic in chickens. This was consistent with other H7N9 viruses that were isolated in 2013 [45,46].

H7N9 viruses are clustered into two main lineages: the Yangtze River Delta (YRD), which is well recognized as the original source of the H7N9 outbreaks, and the Pearl River Delta (PRD), where viruses also originated from the YRD [47]. In our study, phylogenetic data showed that the two H7N9 isolates belonged to the PRD lineage and clustered with human-origin H7N9 that were isolated in 2013. The result was consistent with several studies. Pearl River Delta was the main area from where H7N9 viruses spread to other provinces in China [47,48]. A major reason for this is that the PRD region has extensive and semi-intensive poultry production systems and live poultry markets facilitating the spread of AIVs.

In general, avian-origin H7N9 viruses were lowly pathogenic in mammals without long-term adaptation [39,46]. In our study, the two H7N9 isolates have a similar genetic background, but there were significant differences in pathogenicity in mice (JTC4, MLD_50_ > 10^6.5^ EID_50_; JTC11, MLD_50_ = 10^3.5^ EID_50_). Whole-genome alignment of the two H7N9 isolates was carried out, and only six amino acid differences mapped in five genes, containing PB2, PB1, PA, HA, and NA. The pathogenicity of AIV in mammals depends on several restriction factors, including its ability to bind to and enter cells and replicate its RNA genome within mammalian cells [6,22,33]. Among these differential amino acids, PB2-E627K was recognized as one of the key markers for the virulence of AIVs in mammals. Three independent theories have been proposed. First, PB2-627K can directly increase polymerase activity and then influence AIV replication in mammals [24,49]. Second, ANP32 proteins have been linked to the function of AIV polymerase. Human homologs of ANP32 enhance the activity of polymerases adapted to humans carrying the PB2-627K mutation but do not exert the same effect on polymerases containing the avian-like PB2-627E mutation [50,51]. Additionally, human importin-α1 and -α7 have been previously identified as positive regulators for human-adapted polymerases with PB2-627K, while they do not exhibit an enhancing effect on avian-like polymerases carrying PB2-627E [52,53]. In this study, the ability of JTC11 to virally replicate in mammals and induce innate immune responses and pathogenicity in mice was higher than that of JTC4. PB2-627K may play an important role in it.

In addition, some studies showed that AIVs naturally harbor PB2-627K, which is insufficient to kill mice [17,54,55,56], indicating that it also needs some other mutation synergy to adapt to mammals. The viral polymerase consists of the three subunits: PA, PB1, and PB2 proteins. PB1-V719M and PA-N444D may also contribute to increasing the virulence of H7N9 viruses. The differential amino acid site on the HA protein (V223G) was in the 220-loop of the receptor-binding site (RBS). The RBS is critical for AIV host range restriction [57,58]. Neuraminidase facilitates viral release from cells by removing sialic acid from sialyloligosaccharides on the cell and viral surface. The functional balance between HA and NA is important for AIV’s infection, transmission, and host range [59,60,61,62]. NA-N322S and G389D mutation may affect it and then affect virulence in mice.

## 5. Conclusions

H7N9 is a zoonotic influenza virus with public health significance. Our study showed that in spite of the high similarity of their genetic background, the two H7N9 isolates had different phenotypes. The two avian-origin H7N9 isolates were all lowly pathogenic for chickens and exhibited comparable binding affinity for both avian-type and human-type receptors. In addition, JTC11 can effectively replicate in vitro and in vivo in mammalian cells/tissues and is highly pathogenic for mice, while JTC4 exhibited low pathogenicity. The two H7N9 isolates had six amino acid differences in terms of their whole genome, including the well-known virulence molecular marker PB2-E627K. Our retrospective analysis indicated that mammal-adapted H7N9 virus existed in poultry in 2013, and this may be one of the important reasons for human infection. While there have been no reported human infections with H7N9 in recent years, it remains crucial to maintain rigorous epidemiological surveillance to preemptively address the possibility of any future epidemic.

## Figures and Tables

**Table 1:** Characteristics of two H7N9 viruses isolated from chicken in 2013.

Strains	Abbreviation	Collection Date	Characteristics			
			EID_50_^a^	TCID_50_^b^	IVPI ^c^	MLD_50_^d^
A/Chicken/Eastern China/JTC4/2013	JTC4	2013.09	10^9.167^	10^5.375^	0	>10^6.5^
A/Chicken/Eastern China/JTC11/2013	JTC11	2013.09	10^7.625^	10^6.625^	0	10^3.5^

^a^ EID_50_, 50% egg infectious dose; ^b^ TCID_50_, 50% tissue culture infective dose; ^c^ IVPI, intravenous pathogenicity index (determined in chickens); ^d^ MLD_50_, 50% lethal dose in mice (expressed as the EID_50_ value corresponding to 1 LD50).

**Table 2:** Hemagglutination assays conducted on goose blood cells treated with neuraminidase.

Strains	HA Titer (log2)		Receptor-Binding Preference
	Untreated GRBCs	Treated GRBCs ^a^	
HD05 (H5N1)	8	0	SAα-2,3 Gal
CA04 (H1N1)	8	8	SAα-2,6 Gal
JTC4 (H7N9)	9	9	SAα-2,3 Gal + SAα-2,6 Gal
JTC11 (H7N9)	10	8	SAα-2,3 Gal + SAα-2,6 Gal

^a^ Goose red blood cells (GRBCs) were treated with α2,3-sialidase.

**Table 3:** Amino acid differences between the two avian-origin H7N9 isolates.

Strains	Amino Acid Residue at Indicated Position					
	PB2-627	PB1-719	PA-444	HA-223 ^a^	NA-322	NA-389
JTC4	E	V	N	V	N	G
JTC11	K	M	D	G	S	D

^a^ H3 numbering.

## Data Availability

Data is contained within the article.
